# Comparison of prevalence and severity of asthma among adolescents in the Caribbean islands of Trinidad and Tobago: results of a nationwide cross-sectional survey

**DOI:** 10.1186/1471-2458-5-96

**Published:** 2005-09-14

**Authors:** Michele A Monteil, Gina Joseph, Catherine Changkit, Gillian Wheeler, Robin M Antoine

**Affiliations:** 1 Department of Para-Clinical Sciences, Faculty of Medical Sciences, University of the West Indies, St. Augustine, Trinidad, West Indies; 2 Scarborough General Hospital, Tobago Regional Health Authority, Scarborough, Tobago, West Indies; 3 Department of Mathematics and Computer Science, Faculty of Sciences and Agriculture, University of the West Indies, St. Augustine, Trinidad, West

## Abstract

**Background:**

Asthma is a growing problem in the Caribbean but the prevalence in most islands is unknown and possible inter-island variation in prevalence has not been determined. A nationwide cross-sectional survey was conducted to compare the prevalence of asthma symptoms among high school students in the two islands of the Republic of Trinidad and Tobago.

**Methods:**

Questionnaire and video instruments based on those developed by the International Study of Asthma & Allergy in Childhood (ISAAC) were used to assess asthma prevalence among 6394 children (age range, 11–19 years; mean age, 14.08 yrs) in the second and third years of 35 randomly selected high schools in Trinidad and Tobago. This cross sectional survey was conducted between September and December 2002.

**Results:**

A total of 4988 questionnaires were available for analysis (3519 in Trinidad and 1469 in Tobago). Among respondents from the two islands, there were no significant differences in the prevalence of ever wheezing (24.1% and 24.3% for Trinidad and Tobago, respectively, RR 0.99, 95% CI, 0.90–1.08); wheezing in the previous 12 months (13.1% & 13.4%, RR 0.98, 95% CI 0.84–1.15); a previous or current diagnosis of asthma (12.8% & 13.5%, RR 0.95, 95% CI 0.82–1.12) and night cough in the past 12 months (35.4% & 38.3%, RR0.93, 95% CI 0.86–1.00). However, symptoms of severe asthma were significantly more common among students from Tobago and included having had more than one acute attack in the past year (13.4% & 15.8%, RR 0.85, 95% CI 0.73–1.00, p = 0.0004), night waking as a result of wheeze (7.4% & 10.9%, RR 0.68, 95% CI 0.56–0.83, p < 0.0001) and speech limitation in the past year (5.2% & 8.7%, RR 0.59, 95% CI 0.47–0.74, p < 0.001) Exercise-associated wheezing was also more frequent among Tobagonian adolescents (17.5% & 20.2%, RR 0.87, 95% CI 0.76 – 0.98, p = 0.04).

**Conclusion:**

Self-reported wheeze is common among adolescents in Trinidad and Tobago. Variation in symptoms was found between the two territories; high school students from Tobago, the less industrialized of the two islands, reported more symptoms of severe asthma and exercise-induced wheeze. Difference in the ethnic composition rather than socio-economic factors may be contributing to the observed differences in symptom prevalence.

## Background

Asthma is a common clinical problem in the Caribbean [1,2] and its prevalence seems to have increased since the 1970s. In Barbados for example, between 1970 and 1990, the attendance at the Asthma Bay, an acute asthma management centre at the only public Accident & Emergency (A&E) department on the island increased from 36 to 360 per month despite an increase of only 10% in the island's population during that time [3]. Despite a growing regional problem, there is still very little known about the prevalence of the disease in most Caribbean territories and whether it varies in different islands. There is also little information available about possible environmental and genetic factors that may contribute to the development and exacerbation of asthma in the Caribbean.

The islands of the Republic of Trinidad and Tobago allow a comparison of prevalence and severity of asthma in two differing Caribbean environments. Trinidad & Tobago are located at the southern end of the Caribbean archipelago and are separated by 32 kilometres. Trinidad (4828 km^2,^) is the most industrialised of the Anglophone Caribbean territories and has petrochemical and gas-based industries [4]. In comparison, tourism is the main source of income for Tobago (300 km^2^). Despite the difference in industrialization, socio-economic parameters such as the level of employment are similar in the two territories; Trinidad and Tobago have employment rates of 62.9% and 66% respectively. The average monthly income for Trinidad ranges from 383USD from one rural county to 647USD in an urban centre. The average monthly income in Tobago falls within this range at 433USD (personal communication from Central Statistical Office (CSO) of Trinidad &Tobago, 2005).

The ethnic composition of the two islands differs substantially; Tobago has a predominantly African population which is similar to many of the other smaller Caribbean territories but Trinidad is multi-ethnic with citizens of African, Amerindian, Caucasian, Chinese, South Asian and Mixed ethnicity [5]. Trinidad and Tobago also exhibit geomorphologic differences. The official language in both islands is English.

A nationwide ISAAC-based questionnaire and video survey was conducted among over 6000 adolescents in the two islands to elucidate the prevalence of symptoms of asthma and to determine if differences in symptom prevalence or severity exist. If such differences exist, they may suggest specific environmental or genetic factors that would be explored by further research.

## Methods

### Study design and study instruments

For our nationwide questionnaire and video survey, we used a computerized database, created by the project team, of all public and private high schools in Trinidad and Tobago. The SPSS version 9.0 statistical program (SPSS Inc., Chicago, Illinois, United States of America) was used to generate a random selection of 20% of schools in Trinidad. Schools were approached in the order in which they were selected. Of the 25 Schools selected in Trinidad, 24 (96%) participated. In Tobago, there are only 11 high schools and all agreed to participate (100%).

The questionnaire and video instruments were based on those developed for global use by the International Study of Asthma and Allergies in Childhood [6]. To the "written" ISAAC questions we added a question related to a family history of asthma: Does your father or mother or brother(s) or sister(s) have asthma? Ethnicity of the respondents was assessed by asking them to state if they were one of the following; African (black), Caucasian (white), South Asian (Indian), Chinese, Mixed or Other. Information on participants' ethnicity was obtained since there have been reports elsewhere of racial differences in asthma prevalence and severity and we wished to determine if such differences exist in our multi-ethnic population. The questionnaires are shown in Appendix 1 [see Additional file 1].

Questionnaires for further analysis were obtained from 4988 students in the selected schools. The students were in either Year 2 or Year 3 of secondary school (high school). Children in Trinidad and Tobago typically begin secondary school at age 11–12 years so that students are generally 12–13 years of age in Year 2 and 13–14 years old in Year 3.

All students in these classes were eligible for participation and were provided with information sheets to take home for their parents. Parents were asked to send a note stating that they did not want their child to participate otherwise all students were included in the survey when research staff visited schools. Some children chose not to participate of their own accord. All schools on the two islands were visited by the same two members of the research team. Researchers distributed questionnaires, read through each question slowly and allowed students to complete the questions. Then a 7-minute video showing asthmatics in distress was shown and the video-associated questionnaires ("video" questionnaire) were administered as described above. Forms were collected from students after completion. The research group, because of funding limitations, was unable to determine reasons why students chose not to participate and also if there were differences between students who responded and those who chose not to.

The study was approved by the Ethics Committee of the Faculty of Medical Sciences, University of the West Indies. Permission to visit schools was obtained from the Ministry of Education.

Responses from completed questionnaires were numbered consecutively and entered into computerized databases using Access software (Microsoft Professional 2000, Microsoft Corporation, Redmond, Washington, United States of America)

### Outcome measures

The primary outcome measure was the prevalence of wheeze in secondary school-aged children in Trinidad & Tobago in the 12 months prior to the survey. Secondary outcome measures included the prevalence of symptoms suggestive of severe asthma, exercise induced wheeze and a family history of asthma.

### Statistical analysis

In this study we compared the prevalence of wheeze and symptoms suggestive of severe asthma in high school students in Trinidad and Tobago. Data were analyzed using SPSS (version 9.0) statistical package. The chi-square test (χ^2^) was used to compare the prevalence of symptoms while the significance of relative risks (RR) was assessed with 95% confidence intervals (CIs). Correlation between responses to the "written" and "video" questionnaires was determined by comparing the degree of agreement of positive and negative responses to the following questions: the first two questions of the "written" questionnaire with the first two responses of the "video" questionnaire in relation to Scene 1 on the video; the responses to the questions on wheeze after exercise in the past 12 months (question 7 in "written" questionnaire and question 2 in relation to Scene 2 on the video); the responses to the questions on night cough and night waking in the past 12 months (questions 4 and 8 in the "written" questionnaire and questions 2 in relation to scenes 3 and 4 on the video) [see Additional file 1].

## Results

### Response rates

**Figure 1:**
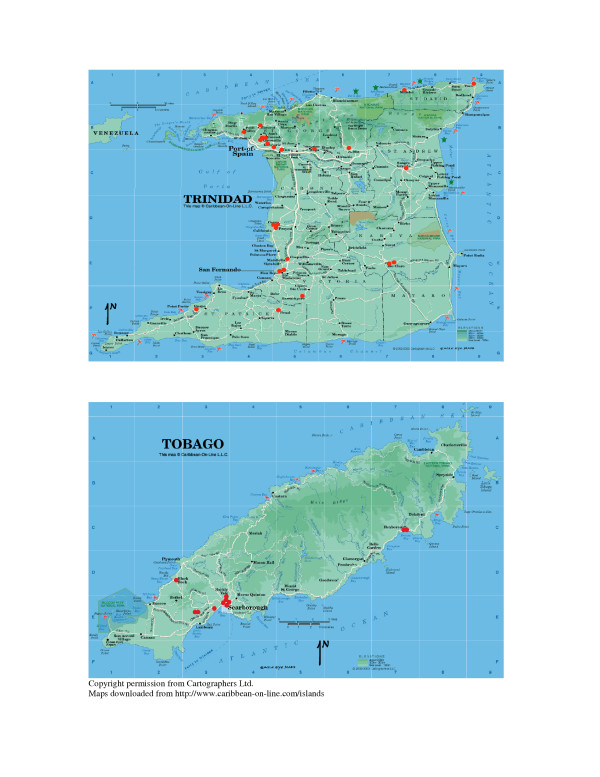
Maps of Trinidad & Tobago showing locations of schools (red dots) that participated in the survey.

A total of 3519 completed questionnaires were retrieved from secondary school children in Trinidad and from 1469 pupils in Tobago. Response rates were 73.3% and 92% for Trinidad and Tobago respectively. These figures correspond to approximately 9% and 92% of all Year 2 and 3 students in Trinidad and Tobago, respectively. There were no negative responses from parents. Student participation at many schools depended on the willingness of students to participate when researchers visited each school. The locations of all participating schools are shown in Figure 1.

### Demographic analysis of participants

Students in the study ranged in age from 11–19 years with average ages of 13.8 and 14.4 years for Trinidad and Tobago respectively. Female participants outnumbered males in both islands (Table 1). In the Trinidadian sample, 36% were South Asian, 35% Mixed, 23.6% African with less than 5% of Chinese, Caucasian or Other ethnicity and 1% were non-responders. On Tobago, there was a predominance of African students (68.5%) with 26% Mixed, 1.4% South Asian and 2% self-reported as being of Chinese, Caucasian or Other race. 2% of Tobagonian students did not respond.

**Table 1:** Summary of the demographic features of study participants

	**Trinidad-Secondary School Students**	**Tobago-Secondary School Students**
**Mean Age, years (range)**	13.77 (11–19)	14.39 (11–18)
**% students of 13–14 years of age**	77.12	70.0
**Male: Female Ratio**	1: 1.47	1.1.23
**Ethnic Composition (%)**		
**South Asian**	36	1.4
**Mixed**	34.8	26
**African**	23.6	68.5
**Caucasian**	2.0	0.4
**Chinese**	0.5	0.2
**Other**	2.0	1.4

### Prevalence of wheeze

848 of 3519 respondents (24.1%) in Trinidad and 357 of 1469 (24.3%) in Tobago reported wheezing in the past (RR 0.99, 95% CI 0.90 – 1.08). Prevalence of wheeze in the previous 12 months was also similar in the two territories; 13.1 % and 13.4% for Trinidad and Tobago respectively, (RR 0.98, 95% CI, 0.84 – 1.15) (Table 2). Four hundred and fifty (12.8%) Trinidadian students and 198 Tobagonian students (13.5%) reported a previous or present diagnosis of asthma (RR 0.95, 95% CI 0.82–1.12). Night cough was the commonest symptom reported by students from both islands. It occurred in 1246 (35.4%) Trinidadian and 563 (38.3%) Tobagonian pupils (RR 0.93, 95% CI 0.86 – 1.00). Exercise-induced wheeze was reported more frequently by students from Tobago (RR 0.87, 95% CI 0.76–0.98, p = 0.04)

**Table 2:** Prevalence and severity of asthma and asthma-associated symptoms among adolescents in Trinidad and Tobago

**Prevalence, %**			
	**Trinidad, n = 3519**	**Tobago, n = 1469**	Relative Risk**, (95% CI)

**% Participation**	73.3	92	
**12 month prevalence**			
**wheeze**	13.1	13.4	**0.98 (0.84–1.15)**
**No. of attacks in past yr**			
**1–3**	10.5	10.9	**0.97 (0.81–1.15)**
**4–12**	1.9	3.0	**0.65 (0.44–0.94)**
**>12**	0.9	1.8	**0.51 (0.31–0.85)**
**Sleep disturbance from wheeze**			
**≤1 per week**	4.7	5.8	**0.81 (0.63–1.05)**
**>1 per week**	2.7	5.1	**0.53 (0.39–0.71)**
			
**Speech limitation**	5.2	8.7	**0.59 (0.47–0.74)**
**Night cough in past year**	35.4	38.3	**0.93 (0.86–1.00)**
**Exercise-induced wheeze**	17.5	20.2	**0.87 (0.76–0.98)**
**Lifetime prevalence**			
**Wheeze**	24.1	24.3	**0.99 (0.90–1.08)**
**Asthma**	12.8	13.5	**0.95 (0.82–1.12)**
**Family History of Asthma**	23.0	13.4	**1.72 (1.49–1.98)**

** Relative Risk of symptom prevalence between Trinidad & Tobago. Tobago is the referent island

Symptoms suggestive of severe asthma were reported more frequently by students from Tobago: 4 or more attacks of wheeze in the past 12 months (99 (2.8%) of 3519 in Trinidad and 71(4.8%) of 1469 in Tobago, RR 0.58, 95% CI 0.43 – 0.79, p = 0.0005); sleep disturbance from wheeze 1 or more times per week in the past year (261 (7.4%) Trinidadian students versus 160 (10.9%) Tobagonian pupils, RR 0.68, 95% CI 0.57 – 0.82, p < 0.0001); speech limitation in the past year (183 (5.2%) Trinidadians students versus 128 (8.7%) students from Tobago, RR 0.60, 95% CI 0.48 – 0.74, p < 0.0001).

### Prevalence of wheeze from video-related questionnaire

A total of 4943 completed video-related questionnaires were available for further analysis, 3489 from Trinidad and 1454 from Tobago. Fewer students reported symptoms of wheeze on the video-related questionnaire: 395 (11.32%) Trinidadian and 250 (17.2%) Tobagonian students reported wheezing in the past and 262 (7.5%) Trinidadian and 179 (12.3%) Tobagonian pupils wheezed in the past 12 months. The prevalence of all symptoms on the video-related questionnaire was significantly higher among Tobagonian students than those from Trinidad (Table 3). There was good agreement between the written and video questionnaire responses in both islands; in Trinidad, there was 64% agreement for positive responses and 78% agreement for negative responses and in Tobago, the corresponding figures were 64% and 76% respectively.

**Table 3:** Comparison of responses to asthma video questionnaire by secondary school students in Trinidad and Tobago

	13–14 yr olds Trinidad % (no.)	13–14 yr olds Tobago % (no.)	Relative Risk** (95% CI)	P value
Wheeze ever	11.32 (395)	17.2 (250)	0.66 (0.57–0.76)	p < 0.0001
Wheeze, 12 mths	7.5 (262)	12.3 (179)	0.61 (0.51–0.73)	p < 0.0001
Wheeze, 1 mth	4.7 (163)	8.7 (126)	0.54 (0.43–0.67)	p < 0.0001
Exercise wheeze ever	21.1 (737)	27.6 (401)	0.76 (0.69–0.85)	p < 0.0001
Exercise wheeze, 12 mt	14.7 (514)	20.2 (293)	0.73 (0.64–0.83)	p < 0.0001
Exercise wheeze, 1 mth	9.1 (316)	14.3 (208)	0.63 (0.54–0.74)	p < 0.0001
Night wheeze ever	7.6 (266)	11.2 (162)	0.68 (0.57–0.82)	p < 0.0001
Night wheeze, 12 mth	3.9 (136)	7.4 (108)	0.52 (0.41–0.67)	p < 0.0001
Night wheeze, 1 mth	2.3 (81)	5.8 (84)	0.40 (0.30–0.54)	p < 0.0001
Night cough ever	24.9 (870)	31.8 (461)	0.78 (0.71–0.86)	p < 0.0001
Night cough, 12 mth	16.0 (558)	21.8 (316)	0.73 (0.65–0.83)	p < 0.0001
Night cough, 1 mth	7.3 (256)	12.9 (187)	0.57 (0.48–0.68)	p < 0.0001
Severe wheeze ever	8.9 (312)	12.6 (183)	0.71 (0.60–0.84)	p = 0.0001
Severe wheeze 12 mth	5.4 (189)	7.9 (114)	0.69 (0.55–0.86)	p = 0.0014
Severe wheeze 1 mth	3.1 (109)	6.0 (87)	0.52 (0.40–0.69)	p < 0.0001

**Relative Risk of symptom prevalence between Trinidad & Tobago. Tobago is the referent island

### Gender difference in prevalence of asthma

In both islands wheeze and a diagnosis of asthma were more common among girls than boys (Table 4). However, this gender difference only achieved statistical significance in the Trinidadian population.

**Table 4:** Prevalence of asthma and wheeze by gender

	**Trinidad, %**	**Tobago, %**
**Asthma Ever**		
Boys	5.4	6.7
Girls	7.4	6.7
	*RR 0.73 (0.56–0.95)	*RR 1.0 (0.68–1.47)
**Wheeze Ever**		
Boys	10.2	11.6
Girls	13.9	12.5
	*RR 0.74 (0.61–0.89)	*RR 0.98 (0.70–1.22)
**Wheeze in past yr**		
Boys	5.3	5.7
Girls	7.8	7.7
	*RR 0.68 (0.53–0.89)	*RR 0.74 (0.50–1.10)

* Relative Risk of symptom between boys and girls (referent) in the individual islands

### Ethnic difference in prevalence of asthma

The island of Trinidad is well-known for its multi-ethnic composition; Africans (39.6%), South Asians (40.3%), Mixed (18.4%) and the rest (1.6%) [5]. An analysis of video responses by self-reported ethnicity from among the cohort of Trinidadian students (Table 5) showed that symptoms were significantly less common among South Asian adolescents compared with those of African or Mixed ancestry.

**Table 5:** Comparison of responses to asthma video questionnaire by secondary school students of different ethnic groups in Trinidad

	13–14 yr olds in Trinidad of African ethnicity % (no.)	13–14 yr olds in Trinidad of South Asian ethnicity % (no.)	13–14 yr olds in Trinidad of Mixed ethnicity % (no.)
Wheeze ever	12 (97)	7.8 (98)* ^	14.5 (175)
Exercise wheeze ever	23 (185)	18.5 (233)**!	23.6 (284)
Night wheeze ever	9.8 (78)	4.5 (59)#!	9.1 (109)
Night cough ever	31.1 (247)	16.5 (207)#!	30.3 (360)
Severe wheeze ever	8.8 (70)	6.6 (83)!	11.5 (137)

Comparison of African and South Asian, * = p < 0.01; ** ; ** = p < 0.05; # = p < 0.0001

Comparison of Mixed and South Asian, ^ = p < 0.0001; ! = p < 0.001

## Discussion

We report on the results of an ISAAC based questionnaire and video survey conducted in the twin island republic of Trinidad and Tobago involving almost 5000 adolescents. As far as these authors are aware, this is the largest survey of its kind conducted in the Anglophone Caribbean. Wheeze in the past was reported by almost a quarter of participants on both islands while wheeze in the past 12 months was reported by about 13% of students in both territories. Symptoms suggestive of severe asthma occurred more frequently in students from Tobago. There was good correlation between the questionnaire and video associated responses in both territories. Night cough was the commonest reported symptom and occurred in over 35% of respondents. Symptoms were reported more commonly by female participants and in the Trinidadian cohort, South Asian students reported fewer symptoms on the video questionnaire.

Wheeze in the past and wheeze in the last 12 months occurred less frequently among adolescents in Trinidad and Tobago compared with previous reports from Barbados; 24.2% and 13.3% respectively for T&T compared with 30.1% and 24.2% for Barbados [1]. The prevalence of wheeze in the last 12 months in Trinidad and Tobago is also lower than that reported by many Central and South American countries [7]. The higher prevalence of wheeze reported from Barbados may reflect an increased awareness of asthma symptoms in that island where there have been very successful public education programmes about the disease for several years [8]. The observed difference in prevalence between the two countries is also in keeping with previous reports of higher prevalence with higher per capita income of a country [9]; GDP per capita of Barbados is 16,200USD compared with 9,600 for Trinidad and Tobago [10].

The frequency of occurrence of symptoms of night cough in the past 12 months (35.4% & 38.3% in Trinidad and Tobago, respectively) far exceeded the prevalence of wheeze. The prevalence of night cough was more akin to that reported for rhinitis symptoms in the last year (33.4% & 30.9% in Trinidad and Tobago respectively) which have been reported elsewhere [11] and raises the possibility that in this population night cough could be due to untreated rhinitis rather than asthma.

Symptoms of severe asthma such as sleep disturbance more than once per week (2.7% in Trinidad & 5.1% in Tobago), speech limitation (5.2% & 8.7% in Trinidad and Tobago respectively) and 4 or more attacks of asthma in the past year (2.8% in Trinidad & 4.8% in Tobago) all occurred more commonly among students from Tobago. Additionally, more students from Tobago responded positively on the video questionnaire which asks for information about more severe asthma [7]. The ISAAC video shows 5 scenarios with young asthmatics at rest but in mild respiratory distress; with exercise induced wheezy breathing; with nocturnal cough; with nocturnal dyspnoea and with severe respiratory distress and wheezing.

The increased prevalence of severe asthma symptoms in Tobagonian adolescents compared to Trinidadian students is unexpected since Tobago has less industrial development and is more reliant on tourism and fishing than Trinidad where there are petrochemical parks and more manufacturing industries. Uneven availability of healthcare may have been one factor which contributed to differences in public awareness, under-diagnosis and management of asthma between the two islands. However, we have calculated similar doctor/patient ratios for the two islands (9.26 doctors/10,000 population in Trinidad and 9.95 doctors/10,000 population in Tobago). These figures are based on manpower figures for 2003/2004 from the Ministry of Health and Tobago Regional Health Authority (personal communication) and population data from the 2000 census [5].

Economic parameters as employment rates and monthly income are similar in Trinidad and Tobago suggesting that such factors may not be risk factors for the higher rate of severe asthma symptoms reported by Tobagonian students. In contrast, student ethnicity could be a contributory factor. In Trinidad, African and Mixed adolescents responded positively more often to video-related questions that did students of South Asian race. Since the video questionnaire seeks information about more severe symptoms, this result suggests that children of African and Mixed race may be experiencing more severe symptoms. The predominant ethnic groups in the Tobagonian cohort were African and Mixed (94.5%) so that race may be a factor in the higher level of positive responses to severe symptoms observed in Tobagonian students. Our findings are in keeping with other reports of race as an important association with severe asthma. Fox Ray and her colleagues [12] in their study of racial and income factors associated with hospital admissions for paediatric and adult asthmatics in California found black patients had a four-fold higher admission rate than other ethnic groups even after controlling for income.

Environmental factors such as environmental tobacco smoking (ETS) furry pets and use of foam pillows [13] have been associated with current wheeze and severe asthma in adolescents. Our survey of asthma did not ask about these environmental triggers. In Trinidad and Tobago, foam pillows are commonly used and about 25% of the adult male population smokes [14] so that these factors could be triggers of asthma among our adolescent population and should be explored in future research. We have previously noted the increased prevalence of asthma symptoms among 6–7 year olds who are exposed to ETS [15]. Furry animals such as cats, rabbits, gerbils and hamsters are not popular in our islands. The most popular pets are dogs which tend to be kept outdoors. We therefore do not anticipate pet exposure to be a risk factor for asthma in our context.

In our study, female students experienced more symptoms of wheeze. Gender difference in symptom prevalence has been reported in some but not all ISAAC surveys among adolescents [16]. We also noted that the difference in asthma symptoms between boys and girls to be significant in Trinidad only. We have no explanation for this but hope to explore this in future research.

## Conclusion

Results from a two island ISAAC questionnaire and video survey among almost 5000 adolescents in Trinidad and Tobago have shown that wheeze in the past 12 months is a common symptoms in the population. Variation in the prevalence of more severe symptoms has been noted between the two islands with the surprising results that students from the less industrialised island of Tobago have a higher prevalence of severe symptoms. The observed difference may be related to genetic factors such as race but environmental and cultural contributory factors cannot be ruled out and merit further research.

## Competing interests

The author(s) declare that they have no competing interests.

## Authors' contributions

MAM designed and coordinated study and wrote this paper

GJ and CC conducted all field work in both islands

GW coordinated the study in Tobago

RMA provided assistance with the statistical analysis

## Pre-publication history

The pre-publication history for this paper can be accessed here:



## Supplementary Material

Additional file 1 Appendix 1: questionnaires on asthma symptoms used in nationwide survey. This file contains the modified ISAAC questionnaires used in the nationwide survey of asthma and allergic symptoms which we conducted in Trinidad and Tobago Click here for file
