# Supplementary material for: Comparison of prevalence and severity of asthma among adolescents in the Caribbean islands of Trinidad and Tobago: results of a nationwide cross-sectional survey
Source: BMC Public Health. 2005 Sep 14;5:96. doi: 10.1186/1471-2458-5-96 (PMC1249582; doi:10.1186/1471-2458-5-96)
Supplement: Additional file 1 — Appendix 1: questionnaires on asthma symptoms used in nationwide survey. This file contains the modified ISAAC questionnaires used in the nationwide survey of asthma and allergic symptoms which we conducted in Trinidad and Tobago [file 1471-2458-5-96-S1.doc]

**Appendix 1: Questionnaires on Asthma Symptoms used in Nationwide Survey**

HEALTH SURVEY OF SCHOOLCHILDREN IN

TRINIDAD AND TOBAGO

JOINT PROJECT OF FACULTY OF MEDICAL SCIENCES-UWI, PAN AMERICAN HEALTH ORGANIZATION (PAHO) & THE ENVIRONMENTAL MANAGEMENT AUTHORITY (EMA)

# INSTRUCTIONS

On this page are questions about your name, school and birth date. Please write your answers to these questions in the space provided.

All other questions require that you tick (✓) your answer in a box. IF YOU MAKE A MISTAKE, put a cross () in the box and tick the correct answer. You should give only ONE TICK FOR EACH QUESTION unless otherwise instructed

**DEMOGRAPHIC QUESTIONS**

Name of School:_____________________________________________

Today’s Date: _______/__________/_________

Day month year

Your name:_________________________________________________

Your date of birth: ________/__________/_________

Day month year

Are you: Male [ ] Female [ ]

Are you: Black [ ] White [ ] Indian [ ] Chinese [ ]

Mixed [ ] Other [ ]

1. Have you ever had wheezing or whistling in the chest at any time in the past?

**Yes 🞏 No 🞏**

**IF YOU HAVE ANSWERED “NO” PLEASE SKIP TO QUESTION 6**

**----------------------------------------------------------------------------------------------------------------------------**

1. Have you had wheezing or whistling in the chest in the last 12 months?

**Yes 🞏 No 🞏**

1. How many attacks of wheezing have you had in the last 12 months?

**None 🞏 1 to 3 🞏 4 to 12 🞏 more than 12 🞏**

1. In the last 12 months, how often, on average, has your sleep been disturbed due to wheezing?

**Never woken with wheezing 🞏**

**Less that one night per week 🞏**

**One or more nights per week 🞏**

1. In the last 12 months, has wheezing ever been severe enough to limit your speech to only one or two words at a time between breaths?

**Yes 🞏 No 🞏**

1. Have you ever had asthma?

**Yes 🞏 No 🞏**

1. In the last 12 months, has your chest sounded wheezy during or after exercise?

**Yes 🞏 No 🞏**

1. In the last 12 months, have you had a dry cough at night, even when you did not have a cold or chest infection?

**Yes 🞏 No 🞏**

1. Does your father or mother or brother(s) or sister(s) have asthma?

**Yes 🞏 No 🞏**

**ISAAC International Video Questionnaire**

**SCENE ONE: The first scene is of a young person at rest.**

1. Has your breathing been like this, at any time in your life?

**YES 🞏 NO 🞏**

**If YES:** has this happened in the past year?

**YES 🞏 NO 🞏**

**If YES:** has this happened one or more times a month?

**YES 🞏 NO 🞏**

**SCENE TWO: The second scene is of two young people exercising. One is in a dark shirt and the other is in a white shirt.**

1. Has your breathing been like the boy’s in the dark shirt during or following exercise at any time in your life?

**YES 🞏 NO 🞏**

**If YES:** has this happened in the past year?

**YES 🞏 NO 🞏**

**If YES:** has this happened one or more times a month?

**YES 🞏 NO 🞏**

**SCENE THREE: The third scene is of a young person waking at night.**

1. Have you been woken at night like this at any time in your life?

**YES 🞏 NO 🞏**

**If YES:** has this happened in the past year?

**YES 🞏 NO 🞏**

**If YES:** has this happened one or more times a month?

**YES 🞏 NO 🞏**

**SCENE FOUR: The fourth scene is also of a young person waking at night**.

1. Have you been woken at night like this at any time in your life?

**YES 🞏 NO 🞏**

**If YES:** has this happened in the past year?

**YES 🞏 NO 🞏**

**If YES:** has this happened one or more times a month?

**YES 🞏 NO 🞏**

**SCENE FIVE: The final scene is of another person at rest.**

1. Has your breathing been like this at any time in your life?

**YES 🞏 NO 🞏**

**If YES:** has this happened in the past year?

**YES 🞏 NO 🞏**

**If YES:** has this happened one or more times a month?

**YES 🞏 NO 🞏**
